# Abacus Training Modulates the Neural Correlates of Exact and Approximate Calculations in Chinese Children: An fMRI Study

**DOI:** 10.1155/2013/694075

**Published:** 2013-10-30

**Authors:** Fenglei Du, Feiyan Chen, Yongxin Li, Yuzheng Hu, Mei Tian, Hong Zhang

**Affiliations:** ^1^Bio-X Laboratory, Department of Physics, Zhejiang University, 38 Zheda Road, Hangzhou 310027, China; ^2^Department of Nuclear Medicine, The Second Affiliated Hospital of Zhejiang University School of Medicine, 38 Zheda Road, Hangzhou 310009, China; ^3^Zhejiang University Medical PET Center, Zhejiang University, 38 Zheda Road, Hangzhou 310009, China; ^4^Institute of Nuclear Medicine and Molecular Imaging, Zhejiang University, 38 Zheda Road, Hangzhou 310009, China; ^5^Key Laboratory of Medical Molecular Imaging of Zhejiang Province, Hangzhou 310009, China

## Abstract

Exact (EX) and approximate (AP) calculations rely on distinct neural circuits. However, the training effect on the neural correlates of EX and AP calculations is largely unknown, especially for the AP calculation. Abacus-based mental calculation (AMC) is a particular arithmetic skill that can be acquired by long-term abacus training. The present study investigated whether and how the abacus training modulates the neural correlates of EX and AP calculations by functional magnetic resonance imaging (fMRI). Neural activations were measured in 20 abacus-trained and 19 nontrained Chinese children during AP and EX calculation tasks. Our results demonstrated that: (1) in nontrained children, similar neural regions were activated in both tasks, while the size of activated regions was larger in AP than those in the EX; (2) in abacus-trained children, no significant difference was found between these two tasks; (3) more visuospatial areas were activated in abacus-trained children under the EX task compared to the nontrained. These results suggested that more visuospatial strategies were used by the nontrained children in the AP task compared to the EX; abacus-trained children adopted a similar strategy in both tasks; after long-term abacus training, children were more inclined to apply a visuospatial strategy during processing EX calculations.

## 1. Introduction

Arithmetical calculation is executed everywhere in our daily life, for example, statistics of population in the government, management of financial affairs in a company, and calculation of the sum of price at a grocery store. These calculations are mainly performed by physical tools or devices (pencil with paper, calculators, and computers). With these tools, complex calculations can be done more precisely and efficiently. However, it is discommodious for everyone to carry a physical device everywhere. Therefore, it is more convenient to calculate by mental calculation.

 Several studies have investigated the cognitive mechanism of exact (EX) and approximate (AP) calculations [1–3]. Within the elementary arithmetic, there are two representative formats corresponding to EX and AP calculations: a language-specific format and a language-independent format. The language-specific format is specified for EX arithmetic which relies on language-based representations, and the language-independent format is specified for AP arithmetic which relies on visuospatial representations [1]. Substantial neuroimaging studies have revealed that the parietal cortex, especially bilateral horizontal segment of the intraparietal sulcus (IPS) and the angular gyrus were potential candidates for neural correlates of mental calculation [1, 2, 4–9]. Furthermore, the left inferior frontal cortex and the angular gyrus were responsible for language-based representations and more activated in EX calculations; the bilateral parietal areas around IPS and left superior prefrontal gyrus were responsible for visuospatial representations and more activated in AP calculations.

**Figure 1:**
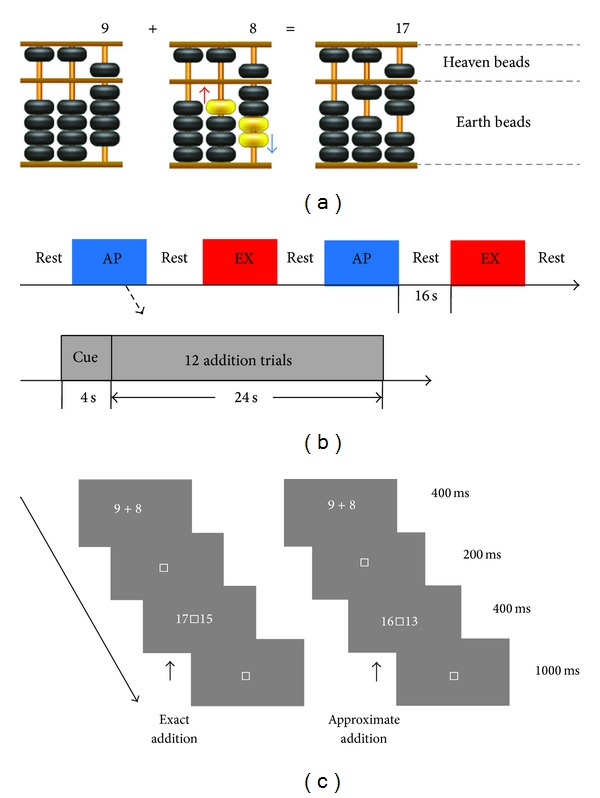
Abacus addition procedure and design of the experimental tasks. (a) An addition example on the abacus (9 + 8 = 17). The left abacus schematic represents the number 9 (one heaven bead equals to 5 and 4 earth beads equal to 4). The middle abacus schematic represents the addition procedure: subtract the complement of the addend to 10 (2 here) by pushing down the 2 yellow beads (near the blue arrow) with the index finger, then add 1 to the tens column by pushing up the yellow bead (near the red arrow) with the thumb. The right abacus schematic represents the result. (b) The task blocks that were used during data acquisition. The blocks were separated by a resting period of 16 s. Each block started with a cue of 4 s to remind the subject of the type of tasks and followed by 12 continuous addition problems. (c) The task design that is used during data acquisition. On each trial, an addition problem is presented for 400 ms and then followed by two alternative answers (also presented for 400 ms). Subjects are instructed to choose either the correct answer (EX task) or the most plausible answer (AP task) as soon as possible.

The other studies found that practice and experience of high-level cognitive skills can change the structure of the cerebral cortex [10–19]. For example, long-term training of abacus-based mental calculation (AMC) does change the neural function [15, 16], structure [17, 18], and the number processing system [19]. AMC is a unique strategy for arithmetic, which can be used to solve calculation problems with exceptional speed and high accuracy [20, 21]. AMC experts can acquire their capabilities after long-term training. Initially, they learn to perform calculations on an abacus (a simple device consisted of beads and rods, and numbers can be represented by the spatial locations of beads) with both hands simultaneously. The procedure of solving a calculation problem by abacus is shown in Figure 1(a). After being familiar with the operation of the abacus, they are instructed to imagine moving beads with actual finger movements on an imagined abacus in minds and watching a real one at the same time. Finally, they calculate via an imagined abacus without moving fingers, as if manipulating a “mental abacus” [15, 20–24]. The existence of the “imaginary abacus” has been validated by previous studies, which indicated that the nonverbal visuospatial cerebral networks play an important role during calculation tasks in abacus experts [15, 16, 23–27]. Recently, a study revealed that “mental abacus” was represented by a column-based model, in which the abacus is split into a series of columns and each column is independently stored as a unit with its own detailed substructure [24].

Positive emission tomography (PET) and functional magnetic resonance imaging (fMRI) studies have attempted to explore the neural correlates of the AMC mechanism in EX calculations [15, 16, 23, 25], which showed that the premotor, frontal, and parietal cortices (especially on the right hemisphere) were involved in AMC. One case study on an adult abacus expert confirmed these special regions by the combined methods of electroencephalography (EEG) and fMRI [27]. Furthermore, a lesion study on a skilled abacus expert manifested the importance of the dorsal premotor and parietal cortex in the superior arithmetic ability of AMC experts [26]. These studies indicated that abacus experts adopted the visuospatial strategy instead of the linguistic strategy when performing EX calculations. 

However, all those studies only focused on the abacus training effect on EX (simple, complex, or both) calculations. To the best of our knowledge, no study has been done to explore the training effects on the AP calculation or the difference between EX and AP calculations. Since the strategy adopted in the EX calculation was changed by long-term abacus training, the difference of the underlying neural correlates between EX and AP calculations might be altered, too. Thus, we hypothesize that: (1) the neural difference between the simple EX and AP calculations should disappear or decline after the abacus training; (2) for simple EX calculations, the mechanism should be changed and more visuospatial representations should be involved after the abacus training.

Since the combined abacus operation and AMC course was instructed only in a few experimental classes at certain elementary schools, starting from Grade 1 to 4, only children were recruited in this study. Thus, our present fMRI study aimed to explore whether and how the abacus training modulates neural correlates of EX and AP calculations in Chinese children.

## 2. Materials and Methods

### 2.1. Subjects

Two groups of children participated in the present study, including an abacus-trained group (10 boys and 10 girls, mean age = 10.60 ± 0.34 years, range: 10.08–11.34 years) and a nontrained group (9 boys and 10 girls, mean age = 10.62 ± 0.31 years, range: 10.12–11.26 years). These two groups were recruited from different classes of the same grade in the same school. At the beginning of this study, all children were randomly divided into two experimental groups. Besides abacus training, all children studied the same curriculum at school. The abacus-trained group was instructed to practice abacus operation and AMC additionally for approximately 3 years and for 2 to 3 hours per week (these combined two practices were named as “abacus training” in this study). In contrast, the nontrained group received no abacus training either at school or after school.

The intelligence quotient (IQ) of each child was assessed by the Wechsler Intelligence Scale for Chinese Children-Revised (WISCC-R) in the same week of MRI study [28]. 

All participants were right-handed urban Chinese children with normal or corrected-to-normal vision and reported no history of neurological or psychiatric disorders. Written informed consent was obtained from each subject and his/her guardian. This study was approved by the Institutional Ethic Committee and the Institutional Review Boards (IRBs) of the Affiliated Hospital of Medical College of Qingdao University.

### 2.2. Tasks

EX and AP addition tasks were applied in this study. The addend ranged from 1 to 9, and the sum ranged from 3 to 17. In the EX addition task, one of the two alternative answers was correct, another was wrong (off by two units at most). In the AP addition task, one of the two alternative answers was a number off by one unit which was the one to be chosen, and another was a number off by at least four units. 

Subjects were instructed to press the left or right button corresponding to the answer they chose. The buttons of correct answers were counterbalanced: half of them were on the left side and another half on the right. Once the answer-choosing screen appeared, subjects were required to push the correct button by their forefingers as fast as possible.

All subjects received 4 blocks of tasks (2 blocks for each). The blocks were separated by a resting period of 16 s. Each block started with a cue of 4 s to remind the subjects of the type of tasks and followed by 12 continuous addition problems. The sequence of blocks was shown in Figure 1(b) and the procedure of a single trial was shown in Figure 1(c).

The experimental task was carried out on a computer using E-prime 1.2 [29]. Stimuli were projected to a screen behind the MR scanner. The subject can see all the tasks by looking at a mirror right over his/her eyes. The MR scanner and the computer which control tasks were synchronized. Reaction time (RT) and accuracy were recorded by the computer.

### 2.3. fMRI Data Acquisition

Data acquisition was performed on a 3.0 T Philips MRI scanner using a standard circularly polarized head coil. For fMRI, a whole brain single-shot gradient-echo echo-planer imaging (EPI) sequence was used, which is sensitive to the blood-oxygen-level dependent (BOLD) contrast. Images were acquired in an interleaved order and approximately parallel to the AC-PC line. Acquisition parameters were as follows: TR = 2000 ms, TE = 30 ms, slice thickness = 4 mm, gap between slices = 0.8 mm, flip angle = 90°, field of view = 230 mm, matrix size = 64 × 64, and slices numbers = 33. Heads of the children were stabilized with a foam cushion to minimize motions during imaging.

### 2.4. Statistical Analysis: Behavioral

Three participants (2 from abacus-trained group, 1 from nontrained group) were excluded due to high error rate (>30%) of the addition tasks. Thus, we got 18 participants for each group.

Trials with RT < 300 ms were discarded from the analysis. Only correct trials were used to calculate the median RTs for each participant under each task condition. These median correct RTs were subjected to a repeated measures of analysis of variance (ANOVA) with task type (the EX and AP task) as a within-subjects factor and group (the abacus-trained and nontrained group) as a between-subjects factor. The same ANOVA was also applied to accuracies in each condition for all participants.

### 2.5. Statistical Analysis: fMRI Data Analysis

Image preprocessing and statistical analysis were carried out with SPM8 (FIL, London, http://www.fil.ion.ucl.ac.uk/spm/), as implemented in MATLAB software (MathWorks, Inc., Natick, MA, USA). In the preprocessing stage, images were (1) corrected for slice timing, (2) spatially aligned to the first volume, (3) normalized to the Montreal Neurological Institute (MNI) standard EPI template with a resolution of 3 × 3 × 3 mm, (4) spatially smoothed with a 6 mm full width at half maximum (FHWM) Gaussian smoothing kernel [30].

After preprocessing, statistical analyses based on general linear model (GLM) and the theory of Gaussian fields [31] were performed at a participant level and at a group level. A fixed effects model was applied to the smoothed data to constitute contrast images for each participant. In this step, an appropriate design matrix was specified and the BOLD signals induced by different experimental conditions were assessed voxel by voxel. Three types of contrast images for different conditions were derived here: the EX, AP, and rest contrast (corresponding to the resting periods between blocks). These contrast images were then submitted to appropriate *t*-tests to identify the activations that were specifically involved in different tasks. 

In order to determine brain activations for different addition tasks, paired *t*-tests were done separately on EX versus rest contrast and AP versus rest contrast in each group. Paired *t*-tests between EX and AP contrasts were also executed to explore the differences in brain activity between the two addition tasks in each group. 

To reveal the differences of brain activity between the abacus-trained and nontrained groups, two-sample *t*-tests were performed between the two groups on both EX versus rest and AP versus rest contrasts.

## 3. Results 

### 3.1. Behavior Result

The accuracies and median correct RTs for each task in both groups were summarized in Table 1. A significant difference was detected in median correct RTs between two tasks: *F*(1, 1) = 189.209, *P* < 0.001, *η* ^2^ = 0.848 (533 ± 86 ms, and 676 ± 124 ms for the EX addition and AP addition task, resp.), which demonstrated that the EX addition task was processed much faster than the AP addition task. The main effect of group and its interaction with task type were not significant (*P* > 0.05).

A significant difference was detected in accuracies between two tasks: *F*(1,2) = 18.671, *P* < 0.001, *η* ^2^ = 0.354 (0.92 ± 0.05 and 0.88 ± 0.07 for the EX addition and AP addition task, resp.), indicating that subjects made more errors in the AP addition task. The difference of accuracies between the groups was also significant: *F*(1, 1) = 7.240, *P* = 0.011, *η* ^2^ = 0.176 (0.91 ± 0.08 and 0.86 ± 0.07 for the abacus-trained and nontrained groups, resp.). Interestingly, unlike the RT analysis, the interaction between group and task type was marginally significant: *F*(1, 2) = 2.681, *P* = 0.111, *η* ^2^ = 0.073. Decomposition into contrasts showed a significant difference in the EX addition task: *F*(1, 1) = 16.276, *P* < 0.001, *η* ^2^ = 0.324 (0.95 ± 0.05 and 0.89 ± 0.04 for the abacus-trained and nontrained groups, resp.), suggesting that nontrained children made more errors in the EX addition task. However, no significant difference was found between the abacus-trained and nontrained groups in the AP addition task (*P* = 0.220).

### 3.2. Brain Activity: General Pattern

**Figure 2:**
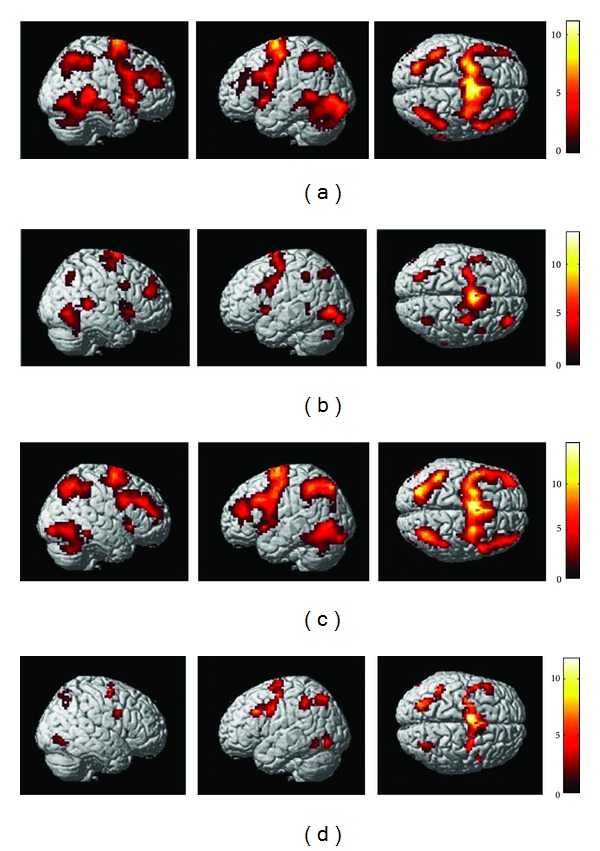
Brain regions activated by the AP and EX addition task in the abacus-trained and nontrained group. (a) The activity pattern revealed by the AP addition task in the abacus-trained group. (b) The activity pattern revealed by the EX addition task in the abacus-trained group. (c) The activity pattern revealed by the AP addition task in the nontrained group. (d) The activity pattern revealed by the EX addition task in the nontrained group. The left column showed the right hemisphere; the middle showed the left hemisphere, and the right showed the vertical view of the brain. (*P* < 0.05, FDR corrected; minimal cluster size = 30 voxels).

Brain regions activated by the AP and EX addition task in the abacus-trained and nontrained groups were investigated separately. A preview of the brain activity was shown in Figure 2. The statistical threshold was set at *P* < 0.05 with false discovery rate (FDR) corrected for multiple comparisons, and a minimal cluster size of 30 voxels was considered to represent regions with significant activations.

In the abacus-trained group, activations elicited by the AP addition task were detected in the supplementary areas (SMA), bilateral precentral sulcus, inferior frontal cortex, bilateral inferior parietal cortices, and bilateral middle temporal and occipital cortices and bilateral insula and thalamus (Figure 2(a)); activations elicited by the EX addition task were detected in the SMA, bilateral precentral sulcus, right middle frontal cortex, bilateral inferior and middle occipital cortices, left inferior parietal cortex, right angular, the bilateral insula and thalamus, right middle and inferior temporal cortices, and the vermis in the cerebellum (Figure 2(b)). The activations for the abacus-trained group were distributed bilaterally in both hemispheres.

In the nontrained group, activations elicited by the AP addition task were detected in the SMA, bilateral inferior and superior parietal cortices, insula, precentral sulcus in the middle frontal cortex, inferior and middle frontal cortices, inferior temporal cortex, thalamus, inferior occipital cortex, and cerebellum (Figure 2(c)); activations elicited by the EX addition task were detected in the SMA, left inferior and superior parietal cortex, left inferior frontal cortex, right angular, left insula, and the bilateral precentral sulcus (Figure 2(d)).

### 3.3. Brain Activity in AP And EX Addition Tasks within Group Comparisons

**Figure 3:**
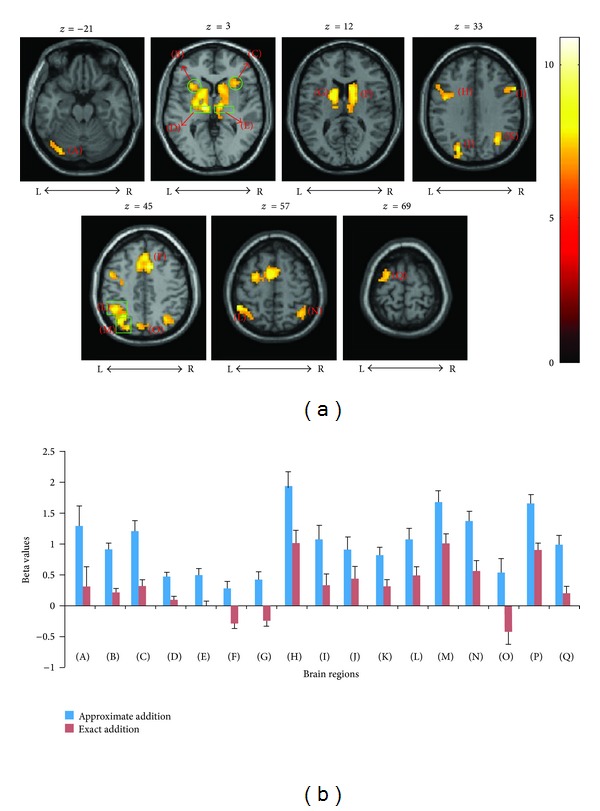
(a) Brain regions activated by the AP addition task (contrasted to the EX addition task) in the nontrained group. (b) Comparison of the mean beta values of these brain regions between two tasks. Uppercase letters in the parenthesis represent these brain regions. (A): cerebellum; (B): left insula; (C): right insula; (D): left thalamus; (E): right thalamus; (F): right striatum; (G): left striatum; (H): left precentral sulcus; (I): right precentral sulcus; (J): left middle occipital lobule; (K): right middle occipital lobule; (L): left inferior parietal lobule; (M): left superior parietal lobule; (N): right angular; (O): left precuneus; (P): SMA; (Q): left superior frontal lobule. (*P* < 0.001, FDR corrected; Voxel sizes > 30).

In the nontrained group, no significant areas were activated in favor of the EX addition task when compared to the AP addition task (*P* > 0.05 uncorrected, cluster size > 20). While activations in favor of the AP addition task were found in the SMA, bilateral inferior parietal cortices, middle occipital cortex, precentral sulcus, left superior parietal cortex, left precuneus, left cerebellum, right angular and the bilateral thalamus, and striatum, with the threshold of *P* < 0.001 (FDR corrected) and a minimal cluster size of 30 voxels (Table 2 and Figure 3).  Figure 3(a) showed brain regions activated by the AP addition task (contrasted to the EX addition task) in the nontrained group; Figure 3(b) showed the mean beta values of activated brain regions in two tasks.

In the abacus-trained group, no significant differences were found in favor of the AP addition task when compared to the EX addition task (*P* < 0.05, FDR corrected). If the statistical threshold was set at *P* < 0.005 (uncorrected) and minimal cluster size as 20 continuous voxels, differences in favor of the AP addition task were found in the left superior frontal cortex (*x*, *y*, *z* = −24, 3, 69), left inferior parietal cortex (*x*, *y*, *z* = −27, −63, 42), and the right middle temporal cortex (*x*, *y*, *z* = 66, −48, −3); In the reverse contrast, only the anterior cingulate cortex (ACC, *x*, *y*, *z* = −3, 30,−6) was activated with the threshold of *P* < 0.01 (uncorrected) and cluster size > 30. This activity pattern suggested that the neural differences between the EX and AP addition tasks declined for abacus-trained children.

### 3.4. Brain Activity in AP and EX Addition Tasks between Group Comparisons

In the AP addition task, more activations were elicited in abacus-trained children, including the bilateral middle temporal cortices (*x*, *y*, *z* = −51, −33, −3 on the left; *x*, *y*, *z* = 51, −18, −12 on the right) and the left precuneus (*x*, *y*, *z* = −3, −39, 60), with a threshold of *P* < 0.001 (uncorrected) and a minimal cluster size of 10 voxels. Conversely, more activations were induced in nontrained children, including the left precentral sulcus (*x*, *y*, *z* = −42, −3, 42) and the SMA (*x*, *y*, *z* = −6, 0, 57), with a threshold of *P* < 0.005 (uncorrected) and a minimal cluster size of 10 voxels.

**Figure 4:**
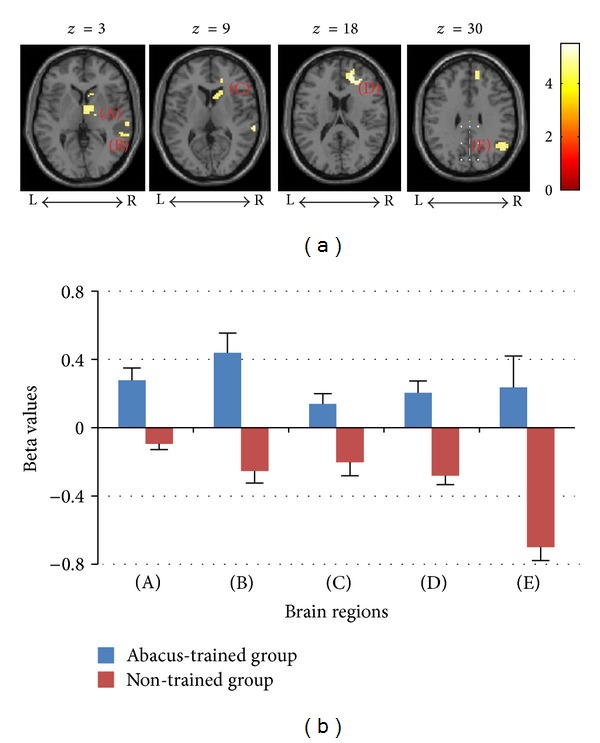
(a) Brain regions activated by the EX addition task in the abacus-trained group (contrasted to the nontrained group). (b) Comparison of the mean beta values of these brain regions between groups. Uppercase letters in the parenthesis represent these brain regions. (A): thalamus; (B): superior temporal lobule; (C): caudate; (D): MPFC; (E): right angular. (*P* < 0.011, FDR corrected; Voxel sizes > 30).

In the EX addition task, more activations were elicited in abacus-trained children, including the medial prefrontal cortex (MPFC), right caudate, right thalamus, right superior temporal cortex, and the right angular gyrus (see Table 3 and Figure 4), with a threshold of *P* < 0.011 (FDR corrected) and a minimal cluster size of 30 voxels.  Figure 4(a) showed brain regions activated by the EX addition task in the abacus-trained group (contrasted to the nontrained group); Figure 4(b) showed the mean beta values of activated brain regions in the two groups. Conversely, more activations were induced in nontrained children, including the left precentral sulcus (*x*, *y*, *z* = −36, −6, 36) and the left middle frontal cortex (*x*, *y*, *z* = −30, 15, 27), with a threshold of *P* < 0.05 (uncorrected) and a minimal cluster size of 30 voxels.

## 4. Discussion

The present study aimed to explore whether and how the long-term abacus training modulates the neural correlates of EX and AP calculations in Chinese children. To address this issue, we compared functional activations between the AP and EX addition task for each group (the abacus-trained and nontrained groups), and we also compared the brain activity patterns between the abacus-trained and nontrained group for each addition task. To the best of our knowledge, this is the first report revealing the specific effect of abacus training on the neural correlates of EX and AP calculations.

The findings confirm our hypothesis. Firstly, the difference between AP and EX addition tasks for abacus-trained children existed only in the FDR-corrected result, demonstrating that the neural correlates underlying the two addition tasks were similar after the abacus training, and the pattern of brain activity during the two addition tasks in the nontrained group was consistent with previous neuroimaging studies [1, 9], revealing that AP arithmetic relied primarily on a quantity representation implemented in visuospatial networks of the left and right parietal lobes. 

Secondly, no differences were detected between the two groups in the AP addition task; while, in the EX addition task, hyperactivity was detected for the abacus-trained children in the right MPFC, right caudate, right thalamus, right superior temporal cortex, and the right angular. These results illustrated that the processing of AP addition task for abacus-trained children was similar to that for nontrained children, but the long-term training changed the neural correlates of the EX addition task; a frontal-temporal circuit was observed, which was also found in a previous study by our group [15].

Furthermore, no differences were detected between the IQs of the two groups, indicating that both groups had comparative intelligence. This supported the argument that the observed differences were likely to correspond to specific neuronal differences.

### 4.1. Behavior Performance

Both of the RT and accuracy analysis showed differences between EX and AP addition tasks. However, the difference between the two groups was only detected for the accuracy of EX addition problems, revealing that abacus-trained children were more accurate than nontrained children in the EX addition task. This was in accordance with previous studies that the abacus experts performed computation tasks with a higher accuracy than control [23, 32]. The vanish of speed effect that the abacus-trained children performed computation tasks with faster speed [23, 32] was probably due to the fact that the abacus-trained children in the present study were not as expert as that in previous studies. Anyway, our results still demonstrated the superior capability of numerical processing for abacus-trained children. A possible account for the better performance of abacus-trained children was that they may employ a different problem-solving strategy induced by the long-term abacus training. After the training, a “mental abacus” was formed in their brains [15, 20], which may be helpful when they performed the arithmetical problems. This “mental abacus” may be the key reason for the improved performance of abacus-trained children in the EX calculation task.

### 4.2. Brain Activations

From the general pattern of brain activities in two addition tasks for both groups, we found that the AP addition task that related brain activations for both groups were distributed bilaterally, while the EX addition that related brain activations were lateralized to the left hemisphere, especially for nontrained children. Moreover, common regions were induced among different addition tasks for both groups, including the SMA, left precentral sulcus (BA 6), insula, IPS, and adjacent parietal areas. Interestingly, the inferior frontal cortex was also activated except in the EX addition task for the abacus-trained group. Basically, these frontal-premotor-parietal areas were specified in numerical representation and calculation, and were consistent with the regions that are engaged in mental calculation tasks in human imaging studies [1, 4–6, 8, 9, 15, 25, 33, 34].

### 4.3. Neural Differences between the AP And EX Addition Task in Each Group

In the nontrained group, common activations across addition tasks covered a cortical network comprised mostly of left-sided superior parietal cortex, SMA, and left precentral sulcus, and these regions were mainly involved in the number of representation and operation tasks [1, 6, 8, 34]. 

A significant difference was detected between the activity patterns of two addition tasks. More activities were induced by the AP addition task, especially in the bilateral parietal areas around the IPS, the precuneus and the superior frontal cortex, indicating that the language-independent visuospatial strategy, were adopted in the AP addition task. This was consistent to the result of previous adult studies [1, 9]. Compatible with previous findings, a greater activation was elicited in the medial frontal cortex (mostly in the pre-SMA), which was associated with the cognitive motor control processes, including sensory discrimination and decision making or motor selection for the action after stimuli [35, 36]. Besides, the medial frontal cortex was also suggested to have a role in mental calculation [37, 38]. The involvement of thalamus and cerebellum which showed a small area of activation in the favor of AP addition task was also detected by a previous study focused on mental calculations [7]. The dorsal basal ganglia, including the caudate and putamen, were known to be critical for procedural memory [39, 40], and this region also played a role in the maintenance of information in working memory in mental arithmetic tasks [41]. The activation of insula also signified the engagement of visuospatial resources when children performed the task [5].

Taken together, these findings provided evidence for the greater involvement of and greater reliance on language-independent strategy and visuospatial working memory in AP addition tasks for nontrained children.

On the other hand, no hyperactivity was detected in favor of the EX addition task. This was dissimilar from previous adults studies in which they found significant activations for EX calculation in the language-dependent areas located in the left hemisphere [1, 9]. One possible reason of the mismatch between the present and previous results may be due to the differences in culture such as educational systems and mathematics learning strategies. For example, Yiyuan Tang et al. [42] detected a functional distinction between native Chinese and English speakers in the brain network that was involved in number representation tasks. Another reason may be due to the strategy and the development of the neural networks adopted by children. A comparative study of children and adults suggested that both verbal and spatial number representations might be involved in AP and EX calculations, and the cerebral networks were still developing around the age of 10 years old [3].

In the abacus-trained group, common activations across addition tasks covered a distributed cortical network that was comprised of bilateral superior parietal cortices, bilateral middle occipital cortices, bilateral inferior temporal cortices, bilateral precentral gyrus, bilateral insula, the SMA, left inferior parietal cortex, and cerebellum. These regions were also found in previous abacus studies [15, 23, 25, 26, 36]. Only slight differences were detected between the AP and EX addition tasks by the uncorrected results; hyperactivity was found in the left superior frontal cortex and left inferior parietal cortex during the AP addition task and in the ACC during the EX addition task. These areas fall outside of traditional perisylvian language areas [43, 44]. The inferior parietal cortex was involved in various visuospatial and analogical mental transformations [45, 46], and the superior frontal-intraparietal network was very important for visuospatial working memory [47]. The ACC that is involved in attentional processes [48] was also found in a previous study in children [49]. These results demonstrated that a similar visuospatial-specific strategy was adopted by abacus-trained children in AP and EX addition tasks, and more visuospatial networks were engaged when they performed AP addition tasks. 

### 4.4. Neural Differences between the Abacus-Trained and Nontrained Group in Each Task

In the AP addition task, no significant differences were found between the two groups in the corrected result, indicating that a similar strategy was adopted in the AP addition task for both groups, whereas, slight differences were detected in the uncorrected result; the spatial related area (left precuneus) and the bilateral middle temporal cortices were more activated in abacus-trained children, while premotor regions (SMA and precentral sulcus) were more activated in nontrained children. Additionally, from the general pattern of the brain activity, we knew that bilateral inferior parietal cortices were activated in both groups during the AP addition task, suggesting the engagement of a visuospatial strategy. These results indicated that a similar but slightly different visuospatial strategy was adopted during the AP addition task in both groups.

In the EX addition task, the abacus-trained group revealed more activations than the nontrained group, including the right MPFC, right caudate, right superior temporal cortex, and the right angular. This activity pattern was specifically lateralized to the right hemisphere, which is consistent to previous abacus studies [15, 23, 25]. The right parietal cortex was suggested to play a role in mental calculation of abacus experts [23]. The right angular was considered to be associated with visuospatial attention [6, 8], and the caudate was suggested to be involved in learning and memory [40]. Besides, the thalamus was also activated. These corticothalamic circuits recruited here may be responsible for the rapid arithmetic processing of abacus-trained children.

More interestingly, the MPFC were mainly included in the default mode network (DMN) which contained a group of areas that exhibit higher metabolic activity at rest than during tasks [50–52]. Additionally, the MPFC was considered to play an important role in cognitive control [53]. This activity pattern indicated that abacus-trained children need fewer resources to accomplish EX addition tasks or the resource allocation was optimized by the long-term abacus training, which might account for the exceptional computational skills of abacus experts. 

All these areas may constitute a circuit involved in the visuospatial-specific retrieval and the processing of imaginary abacus.

## 5. Conclusion

With two matched groups of Chinese children aged about 10 years, the present study investigated the abacus training effect on children's behavioral performance and brain activity pattern during AP and EX addition tasks. Our findings showed that: (1) nontrained children engaged more visuospatial representations in the AP calculation task with contrast to the EX; (2) abacus-trained children adopted a similar strategy for both tasks; (3) after the abacus training, children were more inclined to apply a visuospatial strategy when processing EX problems. For the first time, this study provided evidence for the specific effects of abacus training on EX and AP number processing in children.

However, there were limitations in our study. The lacking of adult contrast made our comparative result between children and adults less persuasive. Furthermore, only one group of abacus-trained children participated in our study; children with different training intensities should be involved in future studies. 

Nonetheless, our results still demonstrated an obvious effect of the long-term abacus training on both of the behavior performance and neural correlates of addition tasks. The present study might be helpful for understanding the neural mechanism of abacus training and also have some positive significance for children's early educations.

## Figures and Tables

**Table 1:** The RTs and accuracies for each task of each group.

Group	Task type	Reaction time (ms)	Accuracy
Abacus-trained	EX addition	526 (101)	0.95 (0.05)
	AP addition	661 (144)	0.89 (0.08)
Non-trained	EX addition	538 (71)	0.89 (0.04)
	AP addition	690 (102)	0.86 (0.05)

Numbers in the parenthesis are standard deviations.

**Table 2:** Brain regions activated by the AP addition task (contrasted to the EX addition task) in the non-trained group.

Regions	Voxel size	BA	Hemisphere	*T* value	MNI coordinate		
					*x*	*y*	*z*
(1) SMA, left precentral sulcus, left superior frontal cortex	696	6/32	L/R	10.72	9	15	48
				8.51	−6	0	57
				8.38	−6	9	51

(2) Thalamus, striatum, insula	953	—	L/R	10.19	−12	−15	3
				7.99	−15	6	9
				7.77	15	12	12

(3) Middle occipital cortex, inferior parietal cortex, superior parietal cortex	454	7/40	L	9.24	−24	−75	30
				8.62	−51	−45	54
				8.48	−30	−63	48

(4) Precentral sulcus	30	9	R	7.96	57	15	36
				5.81	54	6	39

(5) Middle occipital cortex, angular	185	7/40	R	7.40	33	−63	33
				6.85	39	−54	54
				6.62	33	−66	45

(6) Cerebellum	72	—	L	6.62	−33	−81	−21
				6.47	−42	−60	−27
				6.21	−27	−87	−18

(7) Precuneus	43	7	L	6.32	−6	−75	45

BA: Brodmann area. *P* < 0.001 (AP addition contrast versus EX addition contrast, in the non-trained group), FDR corrected.

**Table 3:** Brain regions activated by the EX addition task in the abacus-trained group (contrast to the non-trained group).

Regions	Voxel size	BA	Hemisphere	*T* value	MNI coordinate		
					*x*	*y*	*z*
(1) Medial prefrontal cortex	115	8/9	*R*	5.56	18	42	18
				5.29	27	36	15
				5.05	12	39	36

(2) Superior temporal cortex	31	22	*R*	5.13	66	−33	9
				4.80	66	−24	3
				4.77	63	−42	0

(3) Caudate	48	—	*R*	5.00	15	18	15
				4.43	18	21	−3

(4) Angular	46	39	*R*	4.90	57	−63	27
				4.80	48	−57	27

(5) Thalamus	51	—	*R*	4.84	12	−3	3
				4.67	18	−12	6

BA: Brodmann area. *P* < 0.011 (the abacus-trained group versus the non-trained group, in the EX addition task), FDR corrected.
